# Potential new species of pseudaliid lung nematode (Metastrongyloidea) from two stranded neonatal orcas (*Orcinus orca*) characterized by ITS‐2 and COI sequences

**DOI:** 10.1002/ece3.10036

**Published:** 2023-04-30

**Authors:** Kristina Lehnert, Joy Ometere Boyi, Ursula Siebert

**Affiliations:** ^1^ Institute for Terrestrial and Aquatic Wildlife Research University of Veterinary Medicine Hannover, Foundation Hannover Germany

**Keywords:** Filaroididae, *Halocercus* sp., molecular ecology, odontocetes, phylogeny, Pseudaliidae

## Abstract

Knowledge about parasite species of orcas, their prevalence, and impact on the health status is scarce. Only two records of lungworm infections in orca exist from male neonatal orcas stranded in Germany and Norway. The nematodes were identified as *Halocercus* sp. (Pseudaliidae), which have been described in the respiratory tract of multiple odontocete species, but morphological identification to species level remained impossible due to the fragile structure and ambiguous morphological features. Pseudaliid nematodes (Metastrongyloidea) are specific to the respiratory tract of toothed whales and are hypothesized to have become almost extinct in terrestrial mammals. Severe lungworm infections can cause secondary bacterial infections and bronchopneumonia and are a common cause of mortality in odontocetes. DNA isolations and subsequent sequencing of the rDNA ITS‐2 and mtDNA COI revealed nucleotide differences between previously described Halocercus species from common dolphin (*H. delphini*) and harbor porpoises (*H. invaginatus*) that were comparatively analyzed, pointing toward a potentially new species of pseudaliid lungworm in orcas. New COI sequences of six additional metastrongyloid lungworms of seals and porpoises were derived to elucidate phylogenetic relationships and differences between nine species of Metastrongyloidea.

## Graphical abstract

Rare orca lungworms were molecularly characterized as potential new pseudaliid nematode species using ITS2 and COI nucleotide data. Phylogenetic analyses highlight relationships between metastrongyloid lung nematodes of marine mammals.

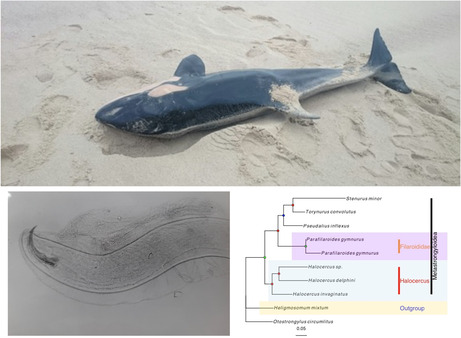

## 1 INTRODUCTION

Information on the parasite fauna of orcas, the prevalence of infections and associated pathology is sparse (Gibson et al., 1998; Raverty et al., 2014, 2020). Only few records on parasites are reported, including species from the gastro‐intestinal tract (six cestode species, anisakid nematodes, two acanthocephalan and two trematode species, Fraija‐Fernández et al., 2016) and records of lungworm infections in two neonatal orcas stranded in Germany and Norway (Reckendorf et al., 2018). The lungworms were found within the bronchi of both individuals, comprising the first record of lungworms in orca (Reckendorf et al., 2018). They were identified as *Halocercus* sp. (Pseudaliidae), which have been described in the respiratory tract of multiple odontocete species (Arnold & Gaskin, 1974; Measures, 2001; Siebert et al., 2006), but morphological identification to species level remained impossible due to the fragile structure and ambiguous morphological features (Pool et al., 2020; Reckendorf et al., 2018). Pseudaliid nematodes are specific to the respiratory tract of toothed whales and comprise multiple species belonging to the superfamily Metastrongyloidea (Anderson, 2000). While other metastrongyloids (Parafilaroididae) infect the airways of pinnipeds and terrestrial carnivores (Anderson, 1982; Lehnert et al., 2010; Rojano‐Doñate et al., 2018), pseudaliids have presumably become extinct in the terrestrial realm (Durette‐Desset et al., 1994). Respiratory nematodes of mongoose are supposed to be the only pseudaliids remaining in terrestrial mammals (Anderson, 1984); however, they were also described as belonging to the Crenosomatidae (Singh & Pande, 1966). Metastrongyloid lungworm infections can have negative impacts on odontocete and phocid health, often causing bronchopneumonia and secondary bacterial infections (Houde et al., 2003; Lehnert et al., 2005; Measures, 2001; Siebert et al., 2006) resulting in mortality. Severe lung nematode burdens result in respiratory distress, obstruction of airways and inhibit the capability to dive and successful foraging (Geraci & Lounsbury, 2001; Rojano‐Doñate et al., 2018; Siebert et al., 2001). Transmission pathways of metastrongyloids in marine mammals are not completely understood. There is evidence of benthic fish intermediate hosts (Dailey, 1970; Houde et al., 2003; Lehnert et al., 2010) in lungworms of pinnipeds and cetaceans, but other studies have indicated that direct infections of *Halocercus* species are possible in bottlenose dolphins (*Tursiops truncatus*, Dailey et al., 1991; Fauquier et al., 2009) and Australian short beaked common dolphins (*Delphinus delphis*) (Tomo et al., 2010). In the two neonatal orcas that were days to weeks old and had been feeding on milk, mature and gravid female lungworms with eggs and larvae in utero were detected in histology (Reckendorf et al., 2018) indicating direct transmission in‐utero or during lactation. In S*krjabinalius guevarai* lungworms infecting striped dolphins (S*tenella coeruleoalba)* in the Mediterranean evidence pointing toward vertical as well as horizontal transmission was found (Pool et al., 2020). Advances with molecular tools now provide unique opportunities to elucidate systematics, ecology, and epidemiology of nematodes and complement, or substitute morphological investigations for identifying and differentiating closely related species when morphological characteristics are not sufficient for species delineation (Mattiucci et al., 2007; Nadler et al., 2005). For the identification of nematode species, the ITS2 region of the internal transcribed spacer provides accurate identification of closely related species (Lehnert et al., 2010; Robles et al., 2014). Additionally, mitochondrial DNA (mtDNA) such as COI evolves rapidly in nematodes and achieves reciprocal monophyly quickly making them suitable for differentiating closely related species (Blouin, 2002).

The aim of this study was to molecularly identify the lung nematodes found in two stranded neonatal orcas by using two gene loci, and differentiate them from related pseudaliid and metastrongyloid nematodes in the respiratory tract of marine mammals.

## 2 MATERIALS AND METHODS

### 2.1 Host animals and parasitology

Details on the strandings, post mortem investigations, and parasite sampling of two neonatal male orcas in 2016 (German North Sea coast) and 2017 (Vesterålen coast, Norway) were previously reported (Reckendorf et al., 2018). Lung nematodes observed in the lungs of both individuals were isolated, cleaned in tap water, and subsequently stored in 70% alcohol for further analyses. Voucher specimens are deposited in the Senckenberg Institute, Forschungsinstitut und Naturmuseum Frankfurt, Frankfurt, Germany (accession nos. German orca: SMF 17057, SMF 17058 / Norwegian orca: SMF 17059, SMF 17060).

Lung nematodes from a common dolphin were collected in 2021 during the necropsy of a solitary individual “Sandy” from the Eckernförder Bay on the Baltic Sea coast of Northern Germany. Voucher specimens are deposited in the Senckenberg Institute, Forschungsinstitut und Naturmuseum Frankfurt, Frankfurt, Germany (accession nos. dolphin lungworm voucher: SMF 17073). Additionally, lungworms belonging to the superfamily Metastrongyloidea collected in 70% Ethanol during routine necropsies of marine mammals at the Institute for Terrestrial and Aquatic Wildlife Research, Büsum in the frame of a stranding network along the German North and Baltic Sea coast line were morphologically identified (Lehnert et al., 2005, 2007; Siebert et al., 2001) and included in the mitochondrial cytochrome c oxidase subunit 1 (COI) and ITS‐2 analyses (Sample info Table 1).

**TABLE 1:** Species included in phylogenetic analyses and their GenBank accession numbers.

Species	Host (scientific name)	Host (common name)	Locality	GenBank accession ITS2	GenBank accession COI
*Halocercus* sp.	*Orcinus orca*	Killer whale	NE Atlantic	OQ379058 (This study)	OQ745800 (This study)
*Halocercus delphini*	*Delphinus delphis*	Common dolphin	Baltic Sea	OQ379059 (This study)	This study
*Halocercus delphini*	*Sternella coeruleoalba*	Striped dolphin	Mediterranean	MN747502 (Pool et al., 2020)	
*Halocercus invaginatus*	*Phocoena phocoena*	Harbor porpoise	NE Atlantic	FJ787301 (Lehnert et al., 2010)	OQ745798 (This study)
*Stenurus minor*	*Phocoena phocoena*	Harbor porpoise	NE Atlantic	FJ787302 (Lehnert et al., 2010)	This study
*Stenurus globicephalae*	*Globicephala melas*	Long‐finnned pilot whale	NE Atlantic	FJ787303 (Lehnert et al., 2010)	
*Torynurus convolutus*	*Phocoena phocoena*	Harbor porpoise	NE Atlantic	AY464532 (Lehnert et al., 2010)	OQ745801 (This study)
*Pseudalius inflexus*	*Phocoena phocoena*	Harbor porpoise	NE Atlantic	FJ767935 (Lehnert et al., 2010)	This study
*Parafilaroides gymnurus*	*Phoca vitulina*	Harbor seal	NE Atlantic	FJ787304 (Lehnert et al., 2010)	This study
*Parafilaroides gymnurus*	*Phoca vitulina*	Harbor seal	NE Atlantic		LT591891
*Otostrongylus circumlitus*	*Phoca vitulina*	Harbor seal	NE Atlantic	AY491979 (Lehnert et al., 2010)	OQ755799 (This study)
*Heligmosomum mixtum*	*Clethrionomys glareolus*	Bank vole	Poland	DQ408626 (Cable et al., 2006)	MN939019
*Hovorkonema variegatum*	*Grus grus*	Common crane	Germany	DQ679968 (Krone et al., 2007)	

### 2.2 Molecular identification / DNA isolation, polymerase chain reaction and sequencing

DNA was isolated from individual nematodes using QIAamp Micro kit (Qiagen, Hilden) according to manufacturer's protocol. Two lungworm individuals of each species were isolated from different hosts. DNA concentrations were determined with the Qubit fluorometer, and DNA integrity with the Thermo Scientific Nanodrop 2000 unit (Peqlab Biotechnologie GmbH).

A region of the ITS‐2 of two lungworm individuals from the dolphin (*n* = 2) and two orcas (*n* = 4) was amplified by polymerase chain reactions (PCRs) using the oligonucleotide primers 5′‐GCA GAC GCT TAG AGT GGT GAA A‐3′ and 5′‐ACT CGC CGT TAC TAA GGG AAT C‐3′ (Lehnert et al., 2010). PCRs were performed in a 50 μL volume containing 25 μL MyTaq Red Mix, 2× (Bioline), 1 μL each of primers at 20 pmol/μL, 5 μL of DNA template and 18 μL of DEPC‐H2O in a peqSTAR 2× Gradient thermocycler (VWR). Cycling conditions were initial denaturation at 95°C for 1 min, followed by 40 cycles of denaturation at 95°C for 15 s, annealing at 60°C for 15 s, and extension at 72°C for 10 s. This was followed by an elongation step at 72°C for 5 min. PCR products were visualized on a 2.0% agarose gel using SYBRSafe DNA Gel stain on a UVP Gelsolo gel documentation system (Analytik‐Jena).

The PCR products were cloned into chemically competent One Shot® *Escherichia coli* cells using pCRTM 4‐TOPO plasmid vector (Invitrogen). At least two clones per individual nematode containing the right insert were inoculated overnight in 5 mL of LBB/Amp liquid. Plasmid DNA was isolated using the PureLink® Quick Plasmid Miniprep (Invitrogen). The rDNA ITS‐2 region was subsequently amplified in PCRs with the isolated plasmid DNA and Sanger sequenced at Microsynth Seqlab (Göttingen). Obtained sequences were aligned by species using CLUSTAL W (Thompson et al., 1994) and intraspecific distances determined in MEGA X (Kumar et al., 2018). Consensus sequence of each species were deposited in GenBank (Accession number: OQ379058–OQ379059) and used in phylogenetic analyses.

Additionally, a partial sequence of the mitochondrial cytochrome c oxidase subunit 1 (COI) mtDNA was amplified from at least two individuals of included lungworm species (*n* = 20, Table 1) using oligonucleotide primers NemF2_t1: ARAGATCTAATCATAAAGATATYGG and NemR2_t1: AWACYTCWGGRTGMCCAAAAAAYCA (Denham et al., 2021; Prosser et al., 2013). Cycling conditions of the PCR were as follows: initial denaturation at 95°C for 1 min, followed by 40 cycles of denaturation at 95°C for 15 s, annealing at 51°C for 15 s, and extension at 72°C for 10 s. The cycling ended with an elongation for 5 min at 72°C. The PCR products were visualized on a 2.0% agarose gel before they were sent to the lab for Sanger sequencing. Sequences were manually examined with SnapGene® Viewer 5.3.2. Intra‐species differences were calculated in MEGA X (Kumar et al., 2018). Consensus sequences of each species were submitted to GenBank and used for phylogenetic analyses.

### 2.3 Phylogenetic analyses

Two sequence datasets (ITS‐2 and COI) were compiled containing new sequences obtained in this study and sequences of other members of Metastrongyloidea available on GenBank (Table 1). Metastrongyoid lungworm sequences were selected according to their availability by browsing GenBank for sequences of Metastrongyloidea and Halocercus (Pseudaliidae) from marine mammal hosts (odontocete and phocid). Five species, *H. invaginatus, H. delphini, H. lagenorhynchi* (unpublished), *H. pingi* and *S. guevarai* ‐ found to be a synonym with *H. delphini* (Pool et al., 2021), were available. Another selection criteria was that ITS‐2 and COI sequence information was available for the selected species. When our Halocercus species did not show any similarity with *H. pingi* when blasted, only sequences generated in this study, or validated by comparisons, like the *H. delphini* sequence by Pool et al. (2021) were included. The two sets of sequences were aligned separately with MAFFT (Katoh & Standley, 2013) in Geneious Prime (version 2022.1.1). Unreliably aligned positions were removed in GBlocks version 0.91b (default parameters, minimum length of a block – “3”, allowed gap position – “with half”) (Castresana, 2000). Nucleotide substitution models were selected for both alignments based on the Akaike information criterion (AIC) implemented in jModeltest2 (Darriba et al., 2012) and used for maximum likelihood and Bayesian inference analyses. Both analyses used HKY + I + G for ITS‐2 and GTR + I + G for COI datasets. Maximum likelihood analyses were conducted using MEGA X software (Kumar et al., 2018) and clade stability was estimated with 1000 bootstrap replications. The Bayesian inference analyses was conducted in MrBayes 3.2.7 (Ronquist et al., 2012). Four Markov chains were run simultaneously for 1,200,000 generations and trees were sampled every 1000th generation for both datasets. An average S.D of split frequencies <0.01 was used as an indication for convergence. A total of 25% of the trees were discarded as burn‐in. Posterior probabilities were calculated as the frequency of clades in the trees sampled after convergence was achieved (Ronquist et al., 2012)

## 3 RESULTS

### 3.1 ITS‐2 sequences

The ITS‐2 sequences from lungworms were between 591 bp (orcas) and 609 bp (dolphin) long. No significant differences were detected between individual specimens of the same lungworm species. The sequences derived from four lungworm specimens of two orca hosts were at least 98% identical. Pairwise genetic distance was between 0.0017–0.0142. When blasted in GenBank, the closest match to the orca lungworm sequence was *Halocercus delphini* with 91.28% identity (Access. No: MN747502).

The ITS‐2 sequences of two lungworm specimens of the common dolphin were 609 bps long and 99.8% identical (Table 2). Pairwise genetic distance was between 0.0000–0.0035. They were shown to be 98.3% identical to *Halocercus delphini* (Accession No: MN747502) isolated from which *Stenella coeruleoalba* in the Mediterranean when blasted in GenBank. The ITS‐2 sequences of lungworm species from orca and dolphin were 93% similar (Table 2), pairwise genetic difference was 0.025. Compared to the dolphin consensus sequence, the orca consensus sequence contained 15 single‐nucleotide polymorphisms‐10 transitions (g.32T>C, g.92A>G, g.106G>A, g.190T>C, g.223T>C, g.333C>T, g.364G>A, g.442C>T, g.469G>A, g.511A>G) and five transversions (g.91T>A, g.188A>T, g.297T>G, g.304A>T, g.393T>A). It also contained 24 nucleotide deletions at five positions (g.159_160del, g.267_274del, g.308_316del, g.456_457del and g.475_477del) and six nucleotide insertions at two positions (g.417_418insTG and g.489_490insGAAA). The sequences of the orca and dolphin lungworms were more similar to each other and to *Halocercus invaginatus* (72.5% and 72.2% respectively) from harbor porpoise than to any other lungworm sequence from odontocete or phocid analyzed (see Table 2 similarity matrix).

**TABLE 2:** Similarity index ITS dataset.

	*Halocercus* sp. (orca)	*Halocercus delphini* (this study)	*Halocercus delphini* (GenBank)	*Halocercus invaginatus*	*Stenurus globicephalae*	*Stenurus minor*	*Torynurus convolutus*	*Pseudalius inflexus*	*Parafilaroides gymnurus*
*Halocercus delphini* (This study)	92.7%								
*Halocercus delphini*	91.5%	98.1%							
*Halocercus invaginatus*	72.5%	72.2%	69.0%						
*Stenurus globicephalae*	46.1%	45.9%	44.5%	43.3%					
*Stenurus minor*	51.6%	51.3%	44.8%	54.7%	59.7%				
*Torynurus convolutus*	50.8%	50.4%	44.4%	53.0%	61.7%	85.9%			
*Psudalius inflexus*	54.9%	53.3%	50.4%	50.3%	60.3%	56.9%	52.6%		
*Parafilaroides gymnurus*	51.2%	51.1%	45.5%	54.5%	44.5%	51.8%	54.1%	49.3%	
*Otostrongylus circumlitus*	43.7%	43.9%	43.5%	43.8%	41.4%	44.8%	45.6%	43.9%	41.6%

### 3.2 COI sequences

COI sequences from eight lungworms were between 618 bp (orca) and 623 bp (*H*. *invaginatus* and *S*. *minor*) long. The sequences from the orcas were at least 98.4% similar, pairwise genetic distance was between 0.00168–0.01022. *Halocercus delphini* sequences were 97% similar with a pairwise genetic distance of 0.02058. The COI sequences from orca and dolphin lungworms were 85% similar, with a pairwise genetic distance of 0.163. They were shown to be 86.4% (orca lungworms) and 84.8% (*Halocercus delphini*) identical to the COI sequence of *Halocercus invaginatus* derived from harbor porpoise. When compared to the COI sequence from dolphin lungworms, the orcas showed 79 single‐nucleotide polymorphisms (44 transitions and 35 transversions) and nine nucleotide deletions. They were more similar to each other and *Halocercus invaginatus* from harbor porpoise than to any other lungworm sequence from odontocete or seal analyzed (see Table 3 similarity matrix). The harbor seal derived *Parafilaroides gymnurus* COI sequence obtained in this study corresponded to 93% identity to a previously published COI sequence from a Dutch North Sea harbor seal found on GenBank (Accession Number: LT591891.1).

**TABLE 3:** Similarity index COI dataset.

	*Halocercus* sp. (*orca*)	*Halocercus delphini*	*Halocercus invaginatus*	*Stenurus minor*	*Torynurus convolutus*	*Pseudalius inflexus*	*Parafilaroides gymnurus*	*Parafilaroides gymnurus* (GenBank)
*Halocercus delphini*	85.6%							
*Halocercus invaginatus*	86.4%	84.8%						
*Stenurus minor*	80.1%	76.6%	80.3%					
*Torynurus convolutus*	84.6%	82.0%	86.7%	85.1%				
*Pseudalius inflexus*	84.5%	82.1%	86.4%	82.3%	88.9%			
*Parafilaroides gymnurus*	80.1%	78.3%	82.3%	79.4%	84.7%	83.9%		
*Parafilaroides gymnurus* (GenBank)	82.4%	82.2%	83.8%	79.9%	86.8%	87.0%	93.2%	
*Otostrongylus circumlitus*	82.1%	81.3%	85.1%	79.4%	84.2%	82.3%	82.6%	83.7%

### 3.3 Phylogenetic analyses

**FIGURE 1:**
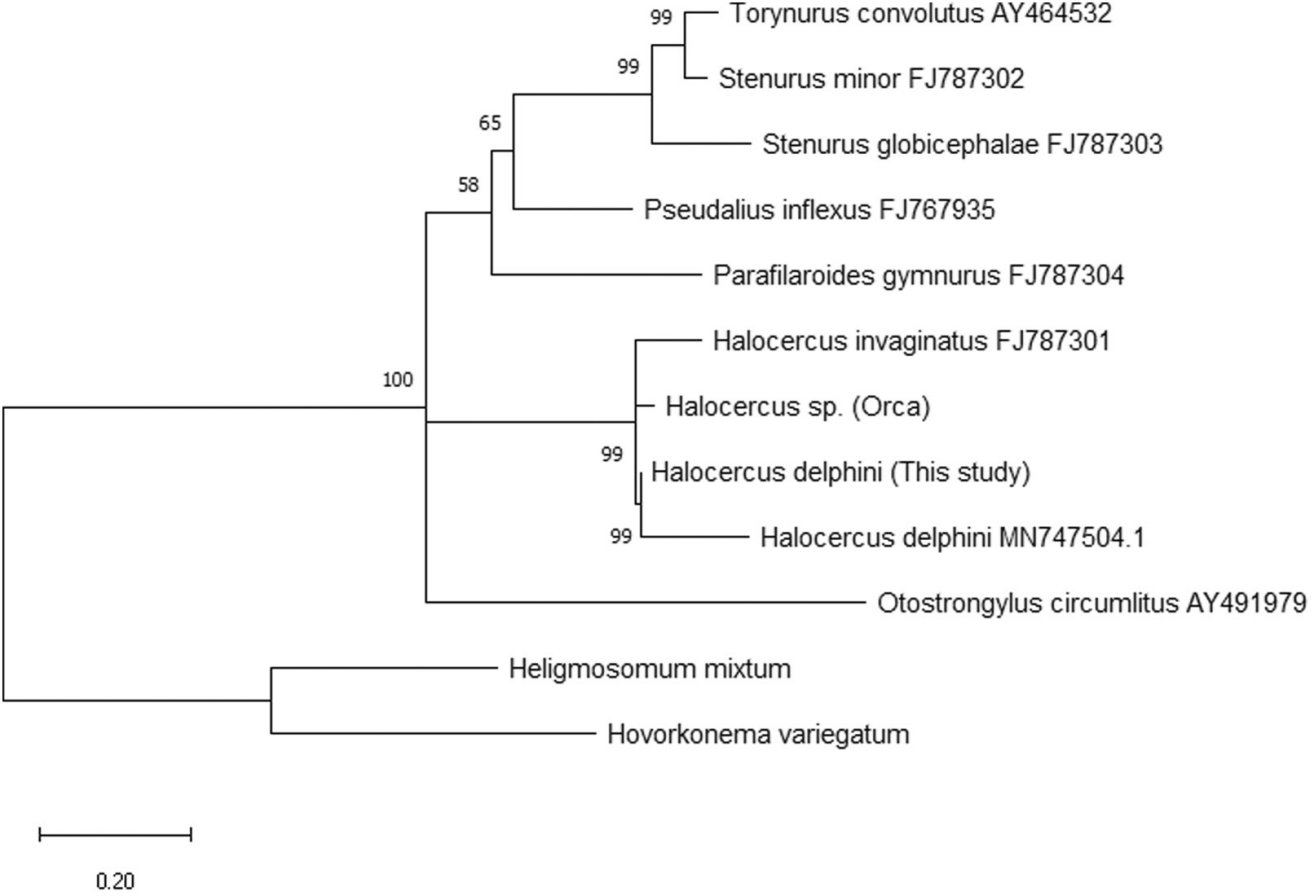
Maximum likelihood phylogenetic tree ITS2 dataset 1000 bootstrap, bootstrap values below 50 are not shown.

**FIGURE 2:**
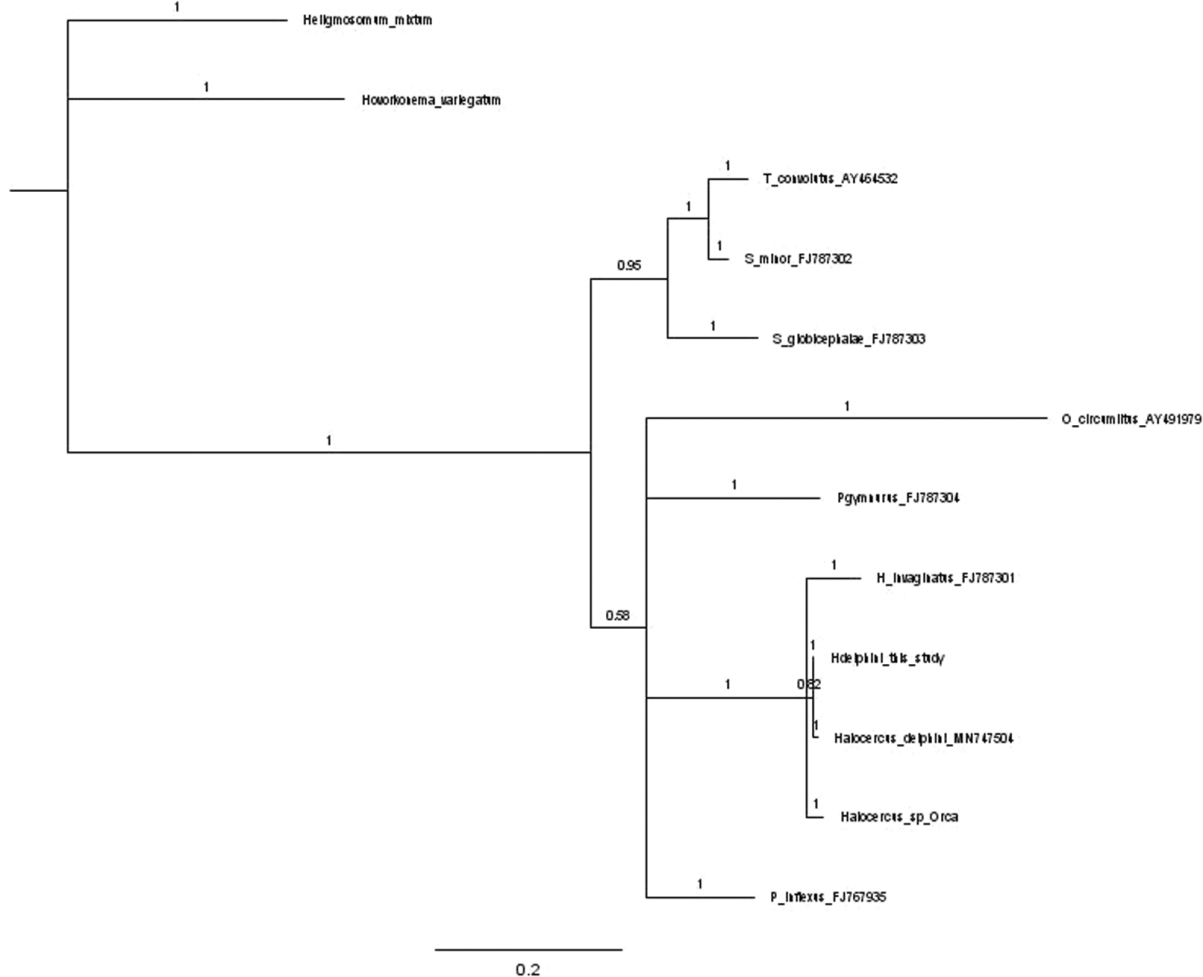
Bayesian phylogenetic tree constructed in MrBayes for the ITS2 dataset with posterior probability values shown.

The ITS‐2 Maximum likelihood tree supported the monophyly of *Halocercus* with a well‐supported clade value (99, Figure 1). The analysis showed a paraphyletic nature of Pseudaliidae with *P*. *gymurus* (Filaroididae) clustering within the clade, although bootstrap support was low. A well‐supported clade containing Metastrongyloidea species also included *O. circumlitus* (Crenosomatidae). Maximum likelihood and Bayesian trees were similar, but in the B.I tree *O. circumlitus*, *P. gymnurus*, *P. inflexus* were placed as sister taxa to the *Halocercus* species. However, the clade was not well supported (0.58, Figure 2).

**FIGURE 3:**
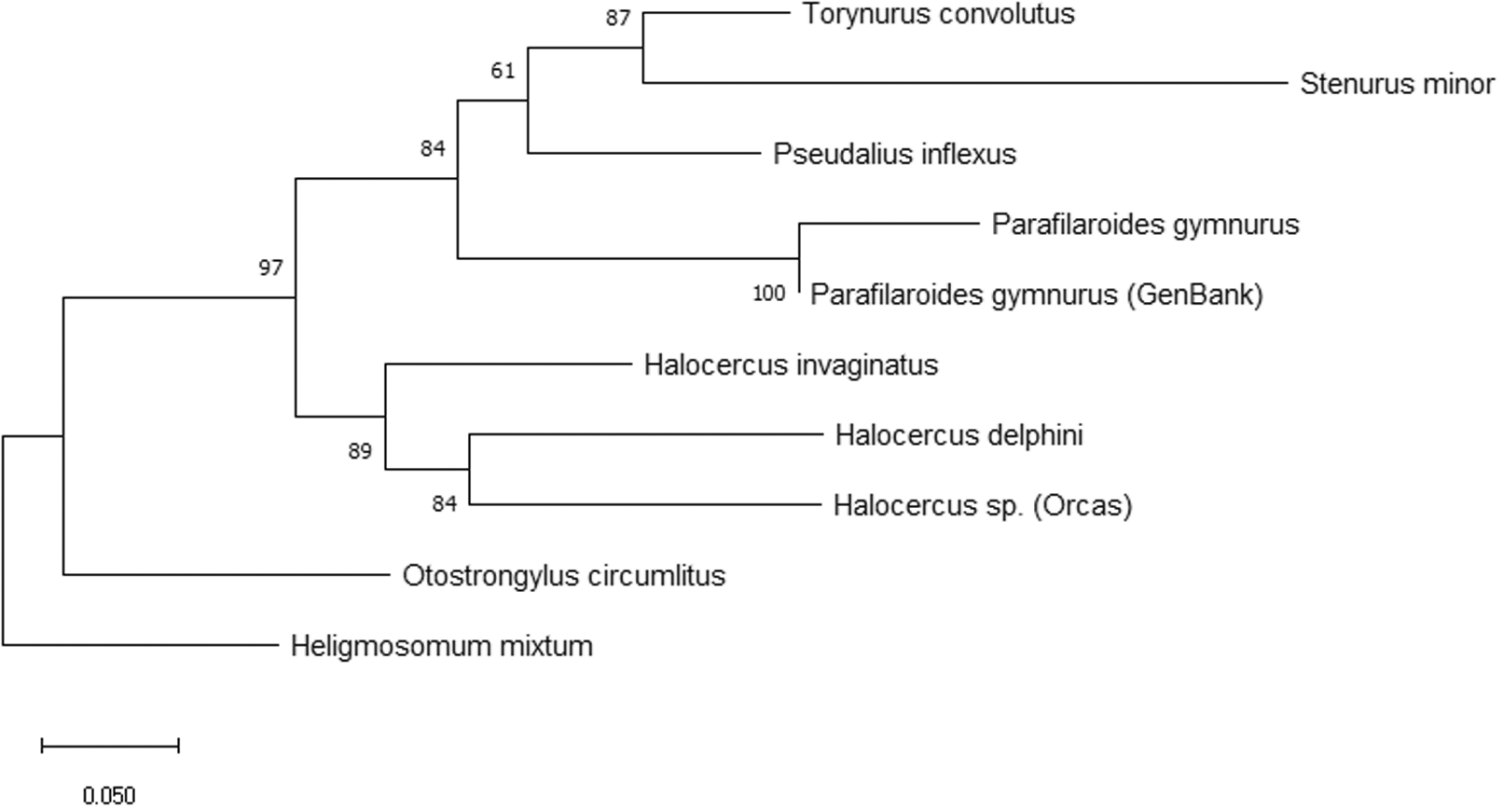
Maximum likelihood phylogenetic tree COI dataset 1000bootstrap, bootstrap values below 50 are not shown.

**FIGURE 4:**
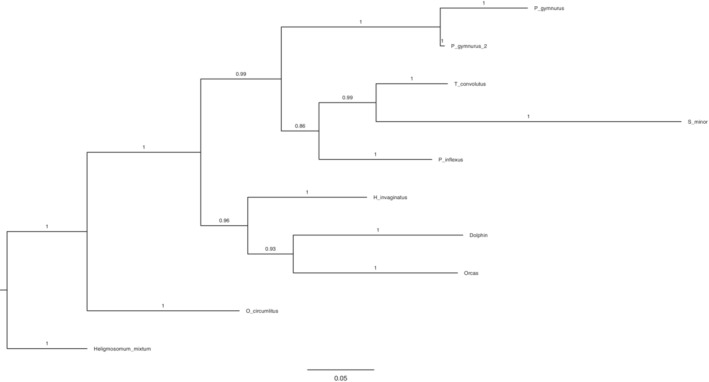
Bayesian phylogenetic tree constructed in MrBayes for Cox1 sequences with posterior probability values shown.

The COI tree was consistent with the ITS‐2 tree. In the COI dataset, the maximum likelihood tree and the Bayesian inference tree are identical (Figures 3 and  4). Paraphyly of Pseudaliidae was shown. *Halocercus invaginatus* is resolved as a sister group to the other *Halocercus* species (89/0.96). *Otostrongylus circumlitus* was placed as a sister group to Pseudaliidae rather than clustered within.

## 4 DISCUSSION

The ITS‐2 and COI nucleotide sequences of orca lung nematodes analyzed in this study were around 600 bps long. The intraspecific differences between ITS‐2 sequences from the two putative lungworm species encountered in common dolphin and orca were 2% and 1%, respectively. This supports the low level of intraspecific variation found in the ITS region of rDNA in nematodes (Campbell et al., 1995; Stevenson et al., 1995), although in comparison, a previous study found no intraspecific variation in ITS‐2 sequences of marine mammal lungworms (Lehnert et al., 2010). The intraspecific differences were considerably lower than the 7% interspecific difference between the two species, reflecting nucleotide dissimilarities typical for separating species (Bazsalovicsová et al., 2010; Blouin, 2002; Robles et al., 2014). This indicates that the orca lungworms should be considered as a different species. The nucleotide data of the ITS‐2 region were supported by COI sequences. The intraspecific differences between sequences from orca, porpoise, and dolphin lung nematodes were between 2 and 3%. This is within the range reported for COI loci in other species (Denham et al., 2021; Elson‐Riggins et al., 2020). The interspecific differences ranged from 15 to 16% in mitochondrial DNA, reflecting sufficient distance to indicate different species (Denham et al., 2021; Hu et al., 2002). Interspecific differences were more prominent in the COI nucleotide than the ITS‐2 sequences, highlighting the fast rate at which mitochondrial DNA accumulates substitutions (Blouin, 2002; Johnson et al., 2003). The observed differences in ITS‐2 and COI sequences between orca lungworm and *Halocercus* sp. individuals of dolphins and porpoises seem sufficient to point toward a previously undescribed pseudaliid lungworm species in orca. Molecular tools are valuable for the unequivocal distinction and the differentiation of the orca lungworm as separate pseudaliid species.

### 4.1 Phylogenetic analyses

For both ITS‐2 and COI sequences, the maximum likelihood and Bayesian analyses resulted in similar trees. In both datasets, the *Halocercus* species are resolved into a well‐supported monophyletic clade. On the phylogenetic tree, *H. invaginatus* is placed as a sister taxon to the lungworm from the orca and *H. delphini*, this phylogenetic relationship is reminiscent of the relationship between the host species (McGowen et al., 2020) suggesting a co‐evolution of parasites and hosts. Interestingly, paraphyly of Pseudaliidae with regard to the filariodid *Parafilaroides gymnurus* was observed in both ITS‐2 and COI sequences in the phylogenetic analysis and is supported by a previous analysis in marine mammal lungworms (Lehnert et al., 2010). *Parafilaroides gymnurus* was originally described as a species of Pseudalius (Railliet, 1899) with an indistinct bursa (Carreno & Nadler, 2003), whereas Filariodidae were characterized by an absent bursa (Anderson, 1978). A close phylogenetic relationship between *P*. *gymnurus* and pseudaliid nematodes of harbor porpoise and pilot whale was observed in ML and Bayesian analysis of the COI sequence dataset, supporting previous analyses on ITS‐2 sequences (Lehnert et al., 2010). The relationship between *P. gymnurus* and Pseudaliidae may be closer than previously assumed. Morphological similarities with *P. gymnurus* concerning size and a reduced bursa can also be found in the pseudaliid *H. invaginatus*. Additionally, both species inhabit the same niche in the lung parenchyma of the respiratory tract in their phocid or odontocete host and cause similar pathologies (Siebert et al., 2001, 2007). The metastrongyloid lung nematodes differ greatly in body size, with the larger species like *P. inflexus* (Pseudaliidae; in harbor porpoise) and *O. circumlitus* (Crenosomatidae; in harbor seals) typically occupying the bronchi and larger airways and blood vessels while the smaller species inhabit bronchioles and smallest like *H. invaginatus* (Pseudaliidae; in harbor porpoise) and *P. gymnurus* (Filaroididae; in harbor seals) the alveoli and lung parenchyma (see e.g. Arnold & Gaskin, 1974; Measures, 2001). The phylogenetic relationships between filaroidid and pseudaliid taxa may appear ambiguous regarding morphological characteristics e.g. an independent loss of the bursa (Carreno & Nadler, 2003), potentially caused by body size or ecological requirements for niche selection. Phylogenetic reconstruction is often complemented by morphological traits; however, loss of defining characteristics may bias the relationships (Bleidorn, 2007; Jenner, 2004). Surprisingly, *S. globicephalae* from the cranial sinuses of pilot whales nested in a clade which otherwise parasitizes porpoises including *S. minor*, *T. convolutus* and *P. inflexus*. Considering the morphology and ecology of the Metastrongyloidea, the three species examined in this study evolved closely with their marine mammal hosts (Dougherty, 1949). Their clear host specificity, despite presumably using the same prey species as intermediate hosts in their life cycles, indicate a strong co‐evolutionary bond. However, it remains to be shown if some pseudaliids can use both direct and indirect transmissions. If the observed species specificity derives from adaptations of the terrestrial ancestors to the marine environment and if it was acquired physiologically or ecologically (Durette‐Desset et al., 1994) remains unclear. The species specificity of three pseudaliid lungworms investigated in five odontocete species from the Mediterranean revealed phylogenetic specificity as well as diet as contributing factors (Pool et al., 2021). A recent study looking at pseudaliid *Stenurus* species in odontocetes off Galicia, Spain, found that different trophic position and niche segregation of hosts may lead to different patterns of specificity (Saldaña et al., 2022).

Analyses of the substitution patterns for mitochondrial genes of nematodes have indicated that they yield useful markers in the genes *cox*1 and *nad*4 for identifying and differentiating cryptic species and for determining relationships of closely related species (Blouin, 2002; Gasser et al., 2008; Hu et al., 2004). Traditionally, the phylogenetic analysis of the taxonomic group Nematoda was based on morphological or ecological characteristics (Hu & Gasser, 2006). However, such studies have been hampered by the paucity of informative morphological characters and the lack of fossil record. Now, and because mt genome sequences have high mutation rates and can provide markers for population genetic studies of parasitic nematodes (Hu & Gasser, 2006), they can complement rDNA nucleotide data and inform about their taxonomy, and life‐history traits. The lungworms characterized in this study are distinct from *Halocercus* species found in other odontocete species. Their speciation may result from ecological segregation of *Orcinus orca* from other delphinids like striped and common dolphins (Delphininae) and pilot whales (Globicephalinae), as well as harbor porpoises (Phocoenidae) geographically, socially, and by dietary preferences over evolutionary timescales. More information about parasite fauna of orca and more sample material from stranded or live sampled animals is needed for further investigations.

## 5 CONCLUSION

Pseudaliid nematodes found in the respiratory tract of two stranded neonatal orcas in northern Europe were characterized using ITS‐2 and COI sequences. Comparative analyses with other marine mammal lungworm species sequences revealed sufficient nucleotide differences to indicate that the orca lungworms could comprise a new, previously undescribed *Halocercus* species. Little is known about parasite fauna of orca and the extraordinary family of the Pseudaliidae. The results underline the value of complementing gene loci nucleotide data for differentiating closely related parasites and elucidating their ecology. Understanding parasite diversity and host specificity is important to assess phylogenetic relationships and ecology of host–parasite dynamics in marine wildlife.

## AUTHOR CONTRIBUTIONS

**Kristina Lehnert:** Conceptualization (lead); data curation (equal); funding acquisition (supporting); investigation (equal); methodology (lead); resources (supporting); supervision (lead); validation (equal); visualization (supporting); writing – original draft (lead); writing – review and editing (equal). **Joy Ometere Boyi:** Data curation (equal); formal analysis (equal); investigation (equal); methodology (equal); validation (equal); visualization (lead); writing – original draft (supporting); writing – review and editing (equal). **Ursula Siebert:** Funding acquisition (lead); resources (lead); writing – review and editing (equal).

## FUNDING INFORMATION

The investigations were partly funded by the Ministry of Energy Transition, Climate Protection, the Environment, and Nature (MEKUN S‐H) and the National Park Service of Schleswig‐Holstein. This Open Access publication was funded by the Deutsche Forschungsgemeinschaft (DFG, German Research Foundation) ‐ 491094227 “Open Access Publication Funding” and the University of Veterinary Medicine Hannover, Foundation. This research received no specific grant from any funding agency, commercial or not‐for‐profit sectors.

## CONFLICT OF INTEREST STATEMENT

The authors declare no conflict of interest.

## Data Availability

Sequences reported in this study are publicly available on Dryad repository: 10.5061/dryad.v15dv421f.
